# Surgical Management of Hereditary Breast Cancer

**DOI:** 10.3390/genes12091371

**Published:** 2021-08-31

**Authors:** Elizabeth R. Berger, Mehra Golshan

**Affiliations:** Department of Surgery, School of Medicine, Yale University, New Haven, CT 06511, USA; mehra.golshan@yale.edu

**Keywords:** hereditary breast cancer, genetic variants, risk reducing surgery

## Abstract

The identification that breast cancer is hereditary was first described in the nineteenth century. With the identification of the BRCA1 and BRCA 2 breast/ovarian cancer susceptibility genes in the mid-1990s and the introduction of genetic testing, significant advancements have been made in tailoring surveillance, guiding decisions on medical or surgical risk reduction and cancer treatments for genetic variant carriers. This review discusses various medical and surgical management options for hereditary breast cancers.

## 1. Introduction

The concept that breast cancer can be inherited and passed from one generation to the next was first described by Paul Broca in 1866. His wife suffered from early age onset of breast cancer, and after studying her family pedigree, he identified four previous generations with breast cancer [1]. This “Broca” report is considered the first of many that has demonstrated the inheritability of breast cancer, and now family history of breast cancer is an established risk factor for the development of the disease [2]. With ongoing research, it is now estimated that up to 15% of patients diagnosed with invasive breast cancer have at least one first-degree female relative with the disease [3]. Furthermore, with the discovery of two major breast cancer susceptibility genes, *BRCA1* and *BRCA2*, in 1994 and 1995, respectively, the association between family history and the presence of inherited genetic events that predispose individuals to breast cancer development was confirmed [4,5,6].

In the last two decades, since the *BRCA* genes were discovered, there have been significant advances in identifying additional germline pathogenic variants in cancer-predisposition genes that have also been associated with an increased risk of breast cancer [7,8,9,10,11,12]. The aggregate prevalence of pathogenic variants in these genes has been estimated to be between 7 and 10% among women with breast cancer [13,14,15,16]. However, these historic estimates are based on high-risk populations (i.e., women with a family history of breast or ovarian cancer, young women with breast cancer and women with TNBC) and thus have uncertain application to the general population [17]. A recent population-based matched analysis by Hu et al. demonstrated that the prevalence of 12 established breast cancer-predisposition genes (*ATM*, *BARD1*, *BRCA1*, *BRCA2*, *CDHS1*, *CHEKs*, *NF1*, *PALB2*, *PTEN*, *RAD51C* and *TP53)* is closer to 5% among women with breast cancer [18]. Improvements in sequencing technology and multigene panel testing have increased the likelihood of detecting these cancer-predisposing variants in not only high-penetrance genes such as *BRCA1/2*, *PALB2*, *CDH1*, *PTEN* and *TP53* but in moderate penetrance genes as well (i.e., *ATM*, *CHEK2* and *NF1).* High penetrance genes confer a 5- to 20-fold lifetime increased risk of breast cancer and moderate penetrance genes confer a 1.5- to 5-fold increased risk [7,11,16,19,20]. Since each of these genes is associated with a different relative risk (RR) of breast cancer, guidance and decision making about screening and prevention strategies such as chemoprevention and prophylactic surgery vary based on the different genetic variants. This article reviews the landscape of inherited mutations of high and moderate penetrance genes and discusses current strategies for clinical management, including surveillance and surgical options for women who carry these genetic variations known to confer a high risk of breast cancer.

## 2. Screening in High-Risk Individuals

Genetic mutations and variants have become more widely known and studied due to increased genetic testing and the increased number of panels used in screening for breast cancer susceptibility. Mutations and variants require nuanced and patient-centric algorithms in terms of management and decision making. Testing allows for the identification of patients at the highest hereditary risk for breast cancer and allows for informed discussions around the prevention and early detection of malignancy [21]. Significant literature has explored primary prevention with risk reducing surgery (RRS) and chemoprevention, as well as secondary prevention strategies such as utilizing magnetic resonance imaging (MRI) in addition to mammography and ultrasound to achieve early detection [22,23]. However, not all genetic variants are created equally. Just as primary prevention strategies such as a risk reducing mastectomy should be considered in patients with *BRCA1/2*, *PTEN* and *TP53* based on the risk they infer and will be discussed later in this article, patients with *ATM*, *CDH1*, *CHEK2, PALB2* and *NF1* should have enhanced screening as data are not sufficient to support a risk-reducing mastectomy in the absence of a strong family history [24]. Various US-based medical societies have recommended breast cancer screening for average risk women to start at various ages. The American College of Radiology (ACR), the Society of Breast Imaging (SBI) and the American College of Obstetricians and Gynecologists (ACOG) all recommend annual mammographic screening starting at the age of 40 [25,26]. The American Cancer Society guidelines state mammography screening should start at the age of 45 based on American Cancer Society guidelines or age 50, based on the United States Prevention Services Task Force (USPSTF) [27,28,29,30,31]. Recommendations for women with a 20% or greater lifetime risk of breast cancer, based on family history, pathogenic variant carriers or other factors such as a previous cancer diagnosis that was treated with radiation therapy to the chest wall, include an annual breast MRI and mammogram beginning at the age of 30 [29,32,33,34].

Screening guidelines for genetic variant carriers differ according to the gene as lifetime risk varies significantly. For women with a *BRCA* variant, the National Comprehensive Cancer Network (NCCN) recommends an annual mammogram, annual MRI and routine clinical breast exams beginning at an early age (25–30) [32,35,36,37]. Because of the relative insensitivity in mammography, due to the underlying breast density of young women at hereditary risk, the benign mammographic appearance of some BRCA-associated breast cancers and the rapid growth rate of these frequently high-grade tumors, MRI and alternating 6-month imaging has emerged as an important tool in breast cancer screening for these women [38]. Multiple studies including Warner et al. and Kriege et al. have demonstrated that MRI has a significantly higher sensitivity and specificity compared to mammograms in women with a high lifetime risk of breast cancer who are screened using both MRI and mammograms concurrently [39,40]. Supplemental MRIs that were also performed annually diagnosed a significantly greater proportion of breast cancers than either mammography and/or ultrasound. Both of these trials also demonstrated that MRI helped identify cancers at an earlier stage, which would suggest MRI screening may result in a survival benefit; however, no randomized control trials have shown this to date. MRI has also been demonstrated as a cost-effective tool when screening high-risk women, as cost-effectiveness increases with breast cancer risk [41,42].

Patients with Li–Fraumeni syndrome (TP53) have an elevated risk of breast cancer, but studies of this population are fraught with ascertainment bias, making precise risk estimation difficult [8,12]. However, breast cancer screening guidelines are similar to those of BRCA carriers, with recommendations of annual clinical breast exams and MRIs to begin at age 20 instead of 25. There are studies that have demonstrated in high-risk women that biannual MRI has a higher sensitivity compared to annual mammographic screening [43,44]. For women with a *PALB2*, *CDH1* or *NF1* mutations, the NCCN recommends an annual mammogram and annual MRI beginning at age 30, unless the age of cancer diagnosis within the family was earlier [45]. For the moderate risk genes, most guidelines suggest screening to begin at age 40 with a mammogram and MRI annually. In many of these moderate penetrance genes, risk is modulated by age, family history and a specific mutation in the particular gene; thus, these factors should be taken into account when discussing surveillance strategies with the individual patient.

## 3. Prophylactic Surgery

Several general strategies are used to reduce cancer risk, morbidity and mortality in women with an increased risk of hereditary breast cancer, including regular imaging screening to detect tumors at an earlier stage, prophylactic surgeries—risk-reducing mastectomy (RRM) and at times, risk-reducing salpingo-oophrectomy (RRSO)—and chemoprevention. In published estimates of cancer risks, there is a considerable range among carriers of different genetic variants; therefore, the role of prophylactic surgery varies from patient to patient. After the initial identification of hereditary breast and ovarian cancer syndrome in 1971, some surgeons began performing prophylactic removals of breast and ovarian tissue in women from families suspected of having this syndrome. This was often performed as a subcutaneous mastectomy, which by today’s standards, would not be considered an adequate removal of all the breast parenchyma [46,47]. However, the efficacy of these procedures was questioned when case reports described the development of breast cancer on the chest wall after mastectomy and peritoneal carcinoma after oophorectomy [48,49]. Published in 1997, the first guidelines for the care of patients with hereditary breast and ovarian cancer syndrome stated that “there was insufficient evidence to recommend for or against prophylactic surgery” [46,50].

In 1990, the *BRCA1* gene was linked for the first time to breast cancer using a large group of early onset breast cancer families and linkage analysis. Mapping of the *BRCA1* gene to chromosome 17q21, cloning of the gene and various truncating mutations were identified in 1994 [51,52]. The ongoing search for additional genes that could be involved in hereditary susceptibility breast cancer families led to the discovery of the *BRCA2* gene in 1995 [4,5]. Subsequently, this was the beginning of the discovery of genetic variants that led not only to a significantly increased risk of breast cancer, but other cancers including ovarian, fallopian tube, colon, melanoma, prostate and pancreatic cancer [53,54,55,56,57,58]. The optimal clinical management of women with the *BRCA1/2* mutations depends on accurate age-specific cancer risk estimates, which can then be used to estimate the absolute risk reduction in preventative strategies and inform decisions regarding the age at which to start surveillance [46,59]. Multiple studies have demonstrated the estimated lifetime breast cancer risk for *BRCA1* carriers to be about 80% and 70% for *BRCA2* carriers [21,59,60,61]. As such, the impact of RRS became the focus of many retrospective and prospective observational studies in the late 1990s and early 2000s.

From 1999 to 2004, four studies were published that compared breast cancer outcomes of women who underwent prophylactic mastectomies to outcomes of women at similar risk who did not undergo surgery [46,62,63,64,65,66,67]. These demonstrated a reduction of 90% or more in the risk of subsequent breast cancer among women who underwent prophylactic surgery with no overall survival differences. There have been additional studies and updated reports that have confirmed these initial observational studies that have led to current position statements by the NCCN saying, “risk-reducing mastectomy provides a high degree of protection against breast cancer in women with BRCA 1 and 2 mutations” and should be discussed on a case-by-case basis with patients who have Li–Fraumeni syndrome or Cowden syndrome [68]. Based on the age at which risk of breast cancer begins, prophylactic mastectomy should not be performed before the age of 25. Furthermore, with the reduced development of breast cancer, breast cancer-specific mortality is also reduced by 90% [69]. A study by Rebbeck et al. demonstrated this 90% risk reduction after bilateral prophylactic mastectomies in BRCA1/2 patients, which was a case-control study, translated into a breast cancer risk of 7% by the age of 70 years [66]. Even though there is a reduction in the development of breast cancer and breast cancer-specific mortality, in the literature, a wide range of uptake of contralateral prophylactic mastectomy by genetic variant carriers is reported. Data suggest a range of 48–90% of women who choose prophylactic surgery versus aggressive surveillance strategies with MRI [70,71,72].

Earlier studies that investigated risk-reducing prophylactic mastectomies in genetic variant carriers discussed simple versus skin-sparing mastectomies, with simple mastectomies remaining the most effective risk-reducing treatment option [73]. Introduction of the nipple sparing mastectomy (NSM) in the 1950s, allowed preservation of the nipple-areolar complex to achieve increased patient satisfaction [74]. Until recently, there was a paucity of data on the use of NSM in high-risk patients, such as BRCA mutation carriers. There were several single-center studies that examined the safety of NSM with small numbers of germline carriers [75,76,77]. Jakub et al., in a multi-institutional setting, demonstrated in 2018 that, of 346 patients who underwent 548 risk-reducing NSMs, there were no ipsilateral breast cancers that occurred after prophylactic NSM with a median follow-up of 34 months. Using risk models for BRCA 1/2 mutation carriers, approximately 22 new primary breast cancers were expected without prophylactic surgery [78]. This has been the largest series of prophylactic NSM in BRCA carriers in the literature to date and, although follow-up remains short, the cumulative evidence supports NSM as an appropriate risk-reducing procedure for patients with genetic variants. A review by Lewis et al. and Rocco et al. discussed and demonstrated the safety of NSM in genetic variant carriers [79,80].

*PALB2* is now considered high risk by many because it confers an RR of breast cancer by more than five-fold. For this high-risk mutation, along with others including *CDH1* and *TP53,* the recommendations are now to consider prophylactic mastectomies for risk reduction [8,81]. Outcomes of bilateral prophylactic mastectomies in patients with moderate penetrance genes are not available; moreover, surgical risk reduction is generally not encouraged based on the level of risk that these mutations impart to women [12]. In comparison, atypical ductal and lobular hyperplasia have a similar increased risk associated with moderate penetrance genes for which prophylactic surgery is rarely discussed or performed. As discussed, these women meet the criteria for high-risk screening with mammograms and MRIs [82].

The risk of ovarian cancer has been well established in the BRCA 1 and 2 patient population, with lifetime estimates ranging from 22% to 65% for BRCA1 and 10% to 35% for BRCA2 mutation carriers [8,59]. Unlike breast cancer screening methodologies, ovarian cancer screening methods using serum markers and/or imaging are largely ineffective [83,84]. The role of RRSO in seven efficacy studies and one meta-analysis has shown not only a reduction in the risk of gynecologic cancers by 95% but also a reduction in the risk of breast cancer by about 50%, most likely through the induction of premature menopause [85,86,87,88]. A recent study by Mavaddat et al. addressed the effect an RRSO had on contralateral breast cancer (CBC) risk in genetic mutation carriers. They demonstrated a statistically significant reduction of CBC after RRSO for BRCA 2 carriers. Furthermore, conducting an oophorectomy before the age of 45 was associated with a greater reduction in cancer risks than conducting an oophorectomy after the age of 45 [89]. RRSO has also been shown to reduce the overall mortality of women by 60% for carriers of the BRCA 1 and 2 mutations [85]. It is thought that there remains a 0.2% annual risk of cancer of the peritoneal lining around the ovaries and fallopian tubes in these women because these tissues cannot be surgical removed during an RRSO; this is compared to a 5–25% risk of developing ovarian cancer in those women not undergoing surgery [85,90]. With a disease such as ovarian cancer, which has both a low detection rate for early stages and high mortality rate for advanced stages, this degree of risk reduction is particularly important [22].

It is often discussed whether a hysterectomy should be performed with salpingo-oophorectomy. There does not seem to be any survival benefit associated with removing the uterus; however, it can simplify future hormonal therapy given to women to reduce the risk of breast cancer or for menopausal symptoms, since Tamoxifen and estrogen replacements are both associated with an increased risk of endometrial cancer [46]. The risks of hysterectomy must also be considered and are well recognized. There are higher rates of infections, hematomas and blood loss when a hysterectomy is performed in combination with salpingo-oophrectomy [22,91,92].

As described, risk-reducing strategies for genetic variant carriers often comprise structured intensified surveillance, chemoprevention at times, lifestyle factors and risk-reducing surgeries. Risk-reducing surgeries can help prevent diseases and thus preserve health and result in additional life years in good health. Recognizing that these aspects of RRS are the most important, there are also data to suggest that RRS in BRCA-mutation carriers is cost effective compared to potentially avoidable cancer treatment costs [93,94]. Schrauder et al. demonstrated that long-term health care costs can be reduced by RRS after genetic testing of BRCA mutation carriers in the German system. In a systematic review, Petelin et al. also demonstrated that combined risk-reducing salpingo-oophorectomy and prophylactic mastectomy resulted in the greatest LE and was cost-effective in most analyses. Despite leading to increased life-expectancy and quality-adjusted life years (QALYs), combined mammography and breast magnetic resonance imaging (MRI) was less likely to be cost-effective than either mammography or MRI alone, particularly for women over 50 and *BRCA2* carriers [95]. However, Gamble et al. demonstrated that risk-reducing mastectomies within 5 years of women after an ovarian cancer diagnosis is not cost effective when compared with breast cancer screening [96]. When thinking about longer-term surveillance, Petelin et al. recently demonstrated that long-term management through a structured multidisciplinary familial cancer service is clinically effective and cost effective for BRCA1/2 carriers [97]. There are various ways to assess cost effectiveness with regard to genetic variant carriers and even genetic testing. It has been shown, however, that high-risk multigene testing for all patients with breast cancer is extremely cost effective compared with testing based on family history or clinic criteria in the United Kingdom and United States health systems [98].

## 4. Breast Cancer in Genetic Mutation Carriers

Approximately 5–10% of breast cancers are associated with a pathogenic germline variant in one of several different genes [99,100]. Over 50% of these pathogenic variants are mutations in the BRCA 1 and 2 genes [51,69,101,102]. Studies of the outcomes of women with these germline mutations have yielded conflicting results, with several reports concluding that women with germline mutations are more likely to die from the disease than women with sporadic breast cancer [103,104,105]. However, studies by Verhoog et al. and Pierce et al. demonstrated similar disease-free and overall survival rates amongst germline carriers and women with sporadic breast cancers that are early stage [106,107]. Mutation carriers’ age at the onset of the disease is 20 years earlier than that of women with sporadic breast cancer, with the age range spanning from patients’ teens to their seventies [69]. BRCA-1 associated breast cancers are more likely to be triple negative (TNBC), whereas BRCA-2-associated breast cancers are often hormonally driven and human epidermal growth factor receptor 2 (HER2)-negative [64,108,109]. Recently, advances in molecular profiling have demonstrated that TNBCs are heterogenous and that a loss-of-function mutation in BRCA1 or BRCA2 represents an opportunity for improved precision treatment [109,110].

### 4.1. Surgical Treatment Strategies

With conflicting data regarding survival rates, questions still remain regarding the best choice of local therapy for genetic variant carriers, more specifically BRCA1 and BRCA 2 carriers, with the understanding that the BRCA1 gene confers a slightly higher risk of cancer development than BRCA2. Age is also an important factor when deciding on local therapy in genetic variant carriers as older women have a smaller additional risk of in-breast tumor recurrence and contralateral breast cancer based on longevity of life alone [111]. It has been well proven that, in women with sporadic breast cancers, breast-conserving therapy (BCT) and radiotherapy (RT) result in an equivalent survival rate to that of mastectomy and are widely used in the management of early stage disease [112,113]. There remain conflicting reports regarding the success of BCT in known carriers of high-risk genes, with some series reporting comparable outcomes to sporadic breast cancer patients, whereas others suggest higher rates of in-breast tumor recurrence (IBTR) [106,114,115,116]. In 2006, Pierce et al. evaluated the outcomes of BRCA 1/2 carriers with breast cancer treated by breast conservation therapy compared with matched sporadic controls that demonstrated similar rates of IBTR between women with mutations and sporadic controls. However, rates of IBTR were twice as high in women who did not undergo an oophorectomy when compared to sporadic breast cancer patients [117]. This study also demonstrated the beneficial effect of tamoxifen use in gene carries who had not undergone an oophorectomy, suggesting a benefit of in-breast tumor control from hormonal interventions. Previous studies had not considered the impact of tamoxifen or risk-reducing BSO on rates of IBTR [106,114,115].

The associations between the reduction of IBTR and contralateral breast cancer events with tamoxifen use and oophorectomy in the study by Pierce et al. are modest in comparison to the 90% or greater reductions observed after bilateral prophylactic mastectomy. A randomized comparison between BCT and RRM is not feasible; however, a meta-analysis by Carbine et al. demonstrated mixed results when examining survival differences in women who had a contralateral prophylactic mastectomy (CRM) after a diagnosis of breast cancer. There were some studies that demonstrated survival differences but, when analyzing the data, the survival advantage was thought to be due to selection bias, with healthier, younger women selecting CRM [118,119,120]. When Metcalfe et al. performed a matched analysis in the BRCA 1/2 population, the survival advantage of CRM was no longer significant [121]. However, it is well known that there is a significantly higher risk of contralateral breast cancer (CBC) in carriers compared with that observed in sporadic control patients [108,111].

### 4.2. Risk of Contralateral Breast Cancer

There are significant differences between the various penetrance genes in relation to the estimation of contralateral breast cancer risk. For women with BRCA1, the cumulative lifetime risk of CBC 20 years after the initial breast cancer diagnosis has been estimated to be approximately 40%. Kuchenbaecker et al. demonstrated that the hazard ratio for CBC declined with increasing age after the first diagnosis of breast cancer [122]. For BRCA 2 carries, the risk has been estimated to be approximately 26% 20 years after the initial diagnosis of breast cancer. Both of these risk estimates have been shown to decrease after a woman undergoes a bilateral salpingo-oophorectomy. Robson et al. demonstrated that women with BRCA 1/2 were significantly more likely to develop CBC 10 years after diagnosis (27% of women with mutations versus 8% of women without) and women not using tamoxifen experienced a higher incidence of CBC, although this was not statistically significant [103]. With regard to moderate penetrance genes, data are limited on the risk of CBC. Patients with a *CHEK2* mutation appear to have a relative risk of CBC of approximately 3.0, although follow-up is limited [12,123]. There are no other studies that report CBC in other moderate penetrance genes such as *ATM* and *NF1*. Various models of germline genetic cancer predisposition would suggest an increased risk of CBC; however, the number of carriers is small and follow-up is limited even in large series from commercial laboratories [7]. This makes risk assessment difficult. Therefore, in the absence of CBC data for these moderate penetrance genes, the discussion of contralateral prophylactic matstectomy should include considerations of the index tumor biopsy and the potential impact of systemic therapy on reducing the risk of CBC. This remains an individualized decision for patients, and breast surgical oncologists play an important role in the decision-making processes regarding these genetic mutation carriers.

## 5. Future Directions

The ongoing development of therapeutic treatments for early stage and metastatic breast cancers, including immunotherapies and poly (ADP-ribose) polymerase inhibitors (PARPi), highlights the importance of genetic testing to help identify patients with breast cancer who might benefit from these various treatments. PARP inhibitors in combination with platinum-based chemotherapies have demonstrated significant promise in the treatment of metastatic BRCA 1/2 mutation carriers [16]. There are ongoing studies investigating these drugs in the setting of early stage breast cancer. Carboplatin has been studied in early stage genetic mutation carriers (GeparSixto/GBG66 and BrighTNess studies) and has demonstrated patients with stage II-III TNBC have significantly higher pathologic complete response (PCR) rates and longer disease-free survival; however, a preplanned sub-analysis of BRCA1/2 status noted that the benefits of adding carboplatin were only significant in patients with wild-type BRCA 1/2 [124]. The CALGB 40,603 study also reported improved PCR with the addition of carboplatin to neoadjuvant therapy in stage II-III TNBC; however, an analysis according to BRCA 1/2 status has not been reported. The role of BRCA testing to guide chemotherapy selection in this setting remains ongoing [125]. Genetic testing, specifically BRCA testing, was previously used in breast cancer patients solely to predict risk of future cancers and guide surgical therapies, but mutation status in all genetic variant-related breast cancers has also increasingly been used to inform patient choices of systemic therapy.

## 6. Conclusions

As genetic testing becomes more prevalent amongst women and indications for panel testing evolve, more confident risk estimates are expected to become available over time, especially for moderately penetrant genes [19]. Initial breast cancer risks and contralateral breast cancer risks are demonstrably higher in the presence of highly penetrant genes when compared to moderately penetrant genes. Surgical risk reduction, for not only breast but ovarian cancer, remains a powerful tool in our armamentarium for many women with genetic variants; however, many questions remain regarding absolute risks of cancer development, contralateral breast cancer risk and ideal timing of prophylactic procedures. Discussions surrounding surgical risk reduction and treatment for breast cancer should be personalized based on risk inferred and a patient’s personal preferences.

## Data Availability

Not applicable.
